# Spaceship Earth Revisited: The Co-Benefits of Overcoming Biological Extinction of Experience at the Level of Person, Place and Planet

**DOI:** 10.3390/ijerph17041407

**Published:** 2020-02-21

**Authors:** Susan L. Prescott, Jeffrey S. Bland

**Affiliations:** 1 The ORIGINS Project, Telethon Kids Institute, University of Western Australia, Perth Children’s Hospital, Nedlands, WA 6009, Australia; 2 inVIVO Planetary Health of the Worldwide Universities Network (WUN), West New York, NJ 10704, USA; jeffbland@plminstitute.org; 3 Personalized Lifestyle Medicine Institute, Tacoma, WA 98443, USA

**Keywords:** planetary health, biodiversity, microbiome, rewilding, dysbiotic drift, mental health, green space, climate change, nature relatedness, food systems, social justice, inflammation, NCDs, mindsets, personalized medicine, narrative medicine, stress, health equity, utopias, environmental health, ecology, extinction of experience, biophilosophy, health promotion, Anthropocene

## Abstract

Extensive research underscores that we interpret the world through metaphors; moreover, common metaphors are a useful means to enhance the pursuit of personal and collective goals. In the context of planetary health—defined as the interdependent vitality of all natural and anthropogenic ecosystems (social, political and otherwise)—one enduring metaphor can be found in the concept of “Spaceship Earth”. Although not without criticism, the term “Spaceship Earth” has been useful to highlight both resource limitations and the beauty and fragility of delicate ecosystems that sustain life. Rene Dubos, who helped popularize the term, underscored the need for an exposome perspective, one that examines the total accumulated environmental exposures (both detrimental and beneficial) that predict the biological responses of the “total organism to the total environment” over time. In other words, how large-scale environmental changes affect us all personally, albeit in individualized ways. This commentary focuses the ways in which microbes, as an essential part of all ecosystems, provide a vital link between personal and planetary systems, and mediate the biopsychosocial aspects of our individualized experience—and thus health—over our life course journey. A more fine-grained understanding of these dynamics and our power to change them, personally and collectively, lies at the core of restoring “ecosystems balance” for person, place and planet. In particular, restoring human connectedness to the natural world, sense of community and shared purpose must occur in tandem with technological solutions, and will enhance individual empowerment for personal well-being, as well as our collective potential to overcome our grand challenges. Such knowledge can help shape the use of metaphor and re-imagine solutions and novel ways for restoration or rewilding of ecosystems, and the values, behaviors and attitudes to light the path toward exiting the Anthropocene.

## 1. Introduction

The term planetary health—defined as the interdependent vitality of all natural and anthropogenic ecosystems (social, political and otherwise [1])—underscores that the health of human civilization is intricately connected to the health of natural systems within the Earth’s biosphere [2]. Ultimately, the health of all “whole” systems depends on the health of every “part” they comprise, with the same principals of interconnectivity applying on each level—from large-scale global systems to the sub-cellular dynamics within each living organism. In this context, the health and resilience of every individual cannot be separated from the health of communities and environments, just as collective personal health determines the health of our societies. Thus, the concept of planetary health blurs the artificial lines between health at scales of person, place and planet, emphasizing the integration of biological, psychological, social and cultural aspects of health in the modern environment, and the need to address these collectively across every level [3].

Given its definition, planetary health addresses the intertwined problems of non-communicable diseases (NCDs), biodiversity losses, climate change, environmental degradation, resource depletion, health inequalities, social injustices, the spread of ultra-processed foods, neoliberalism, over consumption, incivility and other related challenges [4]. Collectively, this quagmire has been referred to as “Anthropocene Syndrome” [5]. According to *Lancet* Editor-in-Chief Richard Horton, planetary health is intended “as an inquiry into our total world. The unity of life and the forces that shape those lives” [6]. Insofar as humans are the generators of Anthropocene Syndrome—and its victims—planetary health ultimately sits within the purview of human attitudes, emotions and behaviors [7]. In other words, it sits with the drivers of personal and collective well-being—where the power for change lies within the reach of each and every individual.

Whatever labels we attach to our current age—Anthropocene, Plantationocene, Capitalocene, etc.—we have to consider holistic exit strategies. In the words of scholar Donna Haraway, “the Anthropocene marks severe discontinuities; what comes after will not be like what came before. I think our job is to make the Anthropocene as short/thin as possible and to cultivate with each other in every way imaginable epochs to come that can replenish refuge” [8]. Hence, with the understanding that not all humans have contributed equally to our current predicament, that is, along lines of socioeconomic privilege/disadvantage, we might look forward to what has been called the “symbiocene” [9].

Although the concept of planetary health has a rich history and has enjoyed growth in recent years [10], the breadth of its interdisciplinary scope has been a barrier to progress. Indeed, Horton has expressed concern that planetary health runs the risk of simply fading into a “recalibrated version of environmental health” [6]. We see the same risk and emphasize the need for an overarching narrative that unites these concepts—one that speaks to the heart as much as the mind—and inspires all-inclusive engagement around a shared goal: the health of our future. 

Volumes of research underscores that we interpret the world through metaphors; indeed, common metaphors are a useful means to enhance the pursuit of personal and collective goals. One enduring metaphor of relevance to planetary health can be found in the concept of “Spaceship Earth”. Here, we revisit this metaphor and place it into the context of emerging research on the exposome and extinction of experience—both psychological and biological—as they relate to health at all scales, from person, to place and planet. 

## 2. Roadmap to the Viewpoint

Here in our Viewpoint article, we will first revisit the early origins and expansion of the Spaceship Earth metaphor; we do this in order to explore its place in the emerging planetary health paradigm. Since the term was popularized by one of the most noted scientists of the 20th century, Rene Dubos, we emphasize the larger context in which Dubos placed the metaphor; that is, an interdisciplinary science that promotes greater inquiry into the biological responses of the whole person to the total environment over time—what we refer to in contemporary lexicon as the exposome. We underscore that Dubos’ scientific specialty was microbiology, and he was among the first to identify the ways in which formerly unseen microbes illuminate the interrelated economy of natural systems, and “give to the phenomenon of symbiosis a significance which transcends analytical biology and reaches into the very philosophy of life” [11].

Since exposome science examines biological responses of the total person to the total environment over time; it forces questions concerning “missing” experiences, or what some researchers have labeled a psychological “extinction of experience” (e.g., less time spent in nature or in contact with biodiversity). We briefly address the research supporting the psychological extinction of experience, and next argue that such behavioral changes are in effect a biological extinction of experience (including missing exposures to diverse microbes) with far-reaching, even trans-generational consequences. We explain that the science exploring the psychological extinction of experience and that behind the biologically oriented biodiversity hypothesis have remained in silos and have not been integrated into the planetary health discourse. The obvious aim of this exercise is to ensure, as *Lancet* Editor-in-Chief Richard Horton stated, that planetary health does not slip back into an environmental health discipline.

With this background in place, that is, the removal of the thin veneer that separates the psychological from the biological, we next argue that the aims of planetary health require a fine-grained understanding of nature relatedness as it is intertwined with the extinction of biological experience. By doing so, we can draw from the metaphor of Spaceship Earth and press for a deeper status report on “spaceship human”, an assessment that acknowledges we are multi-species entities with diverse biota. How does individual and community nature relatedness expand to promote health at all scales? How can distant others, including policymakers and multinational corporations (and their lobbyists and marketing arms), hinder or support the multi-species spaceship human in reaching its fullest potential?

Whether at the scale of a single person, or vast areas of the planet, interventions directed at the “ecological” health are often described as restorative. This can include personalized attempts to restore “missing microbes” (a lack of which may otherwise contribute to planetary non-communicable diseases) or the large-scale introduction of animal predators and large trees. Thus, we next discuss this restoration effort that has been labeled, for better or worse, as “rewilding”. In this section we argue that the success of restoration requires a change in the way we view systems (e.g., food, Educ. ation and political), which either compromise health at scales of the person, place and planet, or ignore the development of an eco-health psyche. Finally, we discuss the utility of Spaceship Earth as a modern-day metaphor. 

At the outset we underscore that the word “total”—whether in Horton’s plea that planetary health moves forward *as an inquiry into our total world*, or the exposome’s total lived experiences of a person meets total environment—is always an approximation, no matter the degree of advancement in scientific assessment techniques. The idea that the “total” lived experiences of a human being (or our total world) can be “known” is not without hubris. Still, there is little doubt that we need to understand more fully the interconnectedness of multiple variables as they impact health at the scales of the person, place and planet over time.

## 3. Spaceship Earth as Metaphor

“This solid globe of ours—this huge rotundity we tread—is a ten-thousand fold more wonderful flying machine than the most daring aeronaut has ever devised or conceived. In the open eye of science it is a huge Spaceship, a vast ether transport bearing the whole human race, at the most incredible speed, swiftly on through the unmeasured spaces of the limitless creation, ignoring all weigh stations and bound for an unknown destiny” Prof. E. Knowlton, 1903 [12].

While it may never be known who coined the term “Spaceship Earth”, it was popularized by NASA’s chief administrator James E. Webb. Addressing the Economic Club of Detroit in March of 1962, Webb exclaimed that “we are all space travelers on the good Spaceship Earth. In another six months we will be 190 million miles away from where we are today” [13]. Due to the price tag of the burgeoning space program and moon shot, broad public support was a necessity. Webb tapped into the collective imagination. His statement was also figurative. With rapid cultural changes, the human experience inside the mid-20th century Spaceship Earth would indeed be “millions of miles” from that travelled by ancestors just decades earlier

Months later, Roger Revelle, an environmental science advisor to the Unites States Secretary of the Interior, underscored the idea of the spaceship community: “What is our Earth, then, but a two-billion-person spaceship hurtling through the void? We face exactly the problems that [astronauts] face. We must not, and indeed we cannot, waste anything. We must somehow learn to live together, to tolerate one another, or else we cannot survive” [14].

The Spaceship Earth metaphor inspired public school science teacher Julius Schwartz and well-known artist Marc Simont to collaborate on a popular children’s book entitled The Earth is Your Spaceship [15]. The book provided an environmental message: “Spaceship Earth has good drinking water...lots of fresh air...Spaceship Earth grows food...food from plants and animals that ride with you through space”. Spaceship Earth has its own music, they wrote, “the whistling of the wind, the roaring of thunder, the pounding of ocean waves” [15]. They encourage a mindful awareness of nature, and although inspiring children to dream that one day they might become an astronaut, they conclude:

“But wherever you go
You will be happy to come back
To your warm,
Green,
Friendly,
Spaceship Earth” [15].

Following this, as the space race peaked in the late 1960s, economists, environmentalists and global health leaders began to use the Spaceship Earth metaphor. One of the better-known scientists to do so was Rockefeller University microbiologist Rene Dubos (1901-1982). In 1969, Dubos wrote a nationally syndicated (United States) newspaper editorial with the Spaceship Earth theme [16], and his keynote lecture at the American Academy of Allergy annual meeting bore the title “Spaceship Earth”. In both venues, Dubos argued for a greater scientific focus on the interrelatedness of the environmental determinants of health and vitality:

“Many of the chronic and degenerative disorders which constitute the most difficult and costly medical problems of our societies have their origins in the surroundings and the ways of life, rather than the genetic constitution of the patient...high priority should be given to the study and control of the forces that affect the quality of human life and its environment, and that are rapidly making the Spaceship Earth a place unfit for human life [17].”

“The term *‘Spaceship Earth’* is no mere catchphrase...despite the irresponsible assertions of a few scientists and imaginative science-fiction writers, we are bound to the Earth by the exigencies of our biological nature...since we make so little effort to investigate the effects of social and technological innovations on human life, we are practicing—not by intention, but irresponsibility—a kind of biological warfare against nature, ourselves and especially against our descendants [16]”

Dubos wrote

With his groundbreaking work in microbiology, Dubos was the ideal messenger to emphasize the need to consider the interrelatedness of ecosystems at all scales. Half-a-century before the gut–brain–microbiome zeitgeist, Dubos used germ-free and specific-pathogen-free mice as a means to clearly demonstrate the effects of stress, dietary factors, maternal care, housing conditions, social interactions and sanitation—including microbial influences—on mammalian health over time (and even over generations) [18,19,20,21]. Today, microbiome science underscores that humans are multi-species entities—and that the total lived experiences accumulated by humans over time is, at least to some extent, mediated by microbial arbitrators.

## 4. The Exposome

“While modern science has been highly productive of isolated fragments of knowledge, it has been far less successful in dealing with complexity...the time has come to give to the study of the responses that the living organism makes to its environment, the same dignity and support which is being given at present to the study of the component parts of the organism” Rene J. Dubos, PhD, 1964 [22].

**Figure 1:**
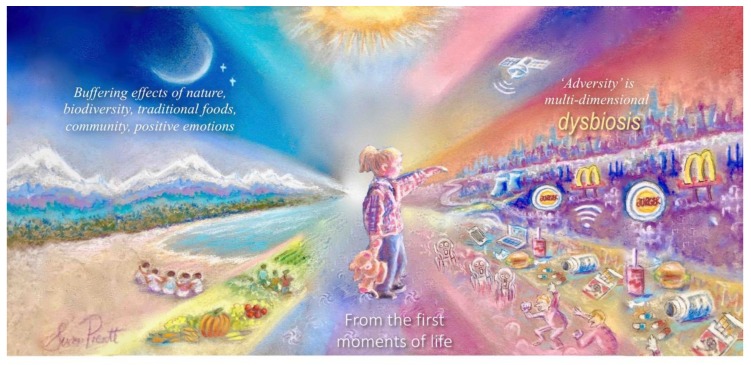
Addressing the multidimensional complexity of the exposome across the life-course: Health depends on (1) minimizing adversity, recognizing that life in distress (dysbiosis) is multidimensional, and (2) promoting the often underestimated value of protective and buffering factors. Both aspects of this challenge may be enhanced by restoring value systems that promote connectivity and responsibility for people, place and planet.

Acknowledging the value of rEduc. tionism and single-variable science, Dubos called for greater inquiry into the biological responses of the total person to the total environment [23,24]. Today, the study of these total accumulated environmental exposures—insofar as they can help predict the biological responses of the “total organism to the total environment” over time—is referred to as the exposome [25]. In effect, exposures (hence the name exposome) are experiences with the potential to be biologically beneficial or detrimental (Figure 1). Thus, adversity in the exposome is not limited to the potential effects of detrimental exposures (e.g., airborne pollutants) but also the absence of protective or buffering factors (e.g., green space), and as with our genome, our exposome is also highly personalized. 

From a life-course perspective, exposome science emphasizes that certain windows of vulnerability (for disease risk) and opportunity (for health promotion) can be identified [26]. Physiological responses are a product of accumulated experiences and may differ, temporally, depending on previous and current experiences; these can include early life stress and nutrition, chronic stressors, neighborhood conditions, social support, the experience of daily hassles and other shifting environmental variables [27,28]. Accordingly, these factors are core to personalized approaches to wellness and disease prevention. Advances in “omics” technologies have afforded researchers the opportunity to simultaneously measure large numbers of biomolecules representing genes, gene expressions, proteins and metabolites. Thus, researchers can identify biological markers of relevance to the total lived experience of individuals, communities and entire populations [28,29,30]. Importantly, these technologies allow individuals to become more engaged in their health, amplifying person-focused action. This may address the limited success of traditional population-based approaches to health, which have arguably not been owned by individuals. 

## 5. Extinction of Experience

“What is the extinction of the condor to a child who has never seen a wren?” [31].

Quite literally, our birds are dying. There are almost 30% fewer birds than in 1970 in North America, with the loss of 3 billion birds over the last 50 years [32], and similar trends in Europe with losses of 421 million birds over 30 years and alterations in relative species abundance [33]. As an indicator of ecosystems health, this is grave cause for grave concern—the “canary in the coal mine” may have already perished.

The Anthropocene is characterized by grotesque levels of species loss; this rapid scale of extinction has been referred to as biological annihilation [34]. The seriousness of biodiversity losses cannot be overstated [35,36,37]. However, insofar as human cognition and behavior is associated with biological annihilation, very little research has been directed at extinction of a different sort. Extinction of experience. 

Recent studies support the idea that adults and children in westernized nations are spending more time indoors [38,39,40]; researchers have demonstrated age-related, cross-generational declines in childhood experiences with nature [41]. Increased engagement with screen-based technology may help explain diminished time outdoors in natural environments [42,43,44,45]. Moreover, cultural products (such as fiction books, film storylines and song lyrics) are trending away from inclusion of nature [46], and the rise of celebrity-oriented mass media has been associated with diminished environmental knowledge [47]. If direct experiences with wild flowering plants are a bellwether of larger interactions with wildlife, it is interesting to note a recent nationwide study in Japan shows that the majority of plant–people interactions are experienced by a small number of people [48]. 

The interrelated findings of greater time spent indoors, widely expanding use of screen-based media and diminished, mindful [49] contact with the natural world are of high-level importance to planetary health. Like miners unaware of poisonous gas, the Anthropocene may be obscuring individual and collective awareness of lost experiences; this extinction of experience is described as the loss of direct, personal, cognitive-emotional contact with plant and animal species, and other elements of the natural world [50,51]. 

There are many pressing research questions surrounding the extinction of experience. Since emerging research indicates that time spent in nature in early life predicts later-life engagement in nature-based activities [52,53,54,55] and subsequent pro-environmental attitudes (and quite likely, pro-environmental behaviors) [56,57], a greater understanding of experiential losses in the context of sustaining life on Spaceship Earth seems urgent. In a self-reinforcing way, experience within natural environments may help develop an emotional connectedness with nature and an enduring “environmental identity” [58,59]. Children with greater experiential nature exposure are more likely to react to signs of environmental harm [60] and protect the environment in the future [56,57]. As we consider that advocacy and action towards planetary health are driven by one’s personal experience, what could be more important?

## 6. Extinction of Biological Experience

The concept of extinction of experience is largely housed in the psychological realm of scientific research; as mentioned, the primary focus is the critical understanding of the ways in which diminished contact with natural environments and biodiversity influence emotions, cognition and behavior. Running in parallel with this line of inquiry is a largely separate body of research which examines the biological consequences of loss of “experience” (the often-used term is exposure) with biodiversity in the context of immune responses and other aspects of human physiology.

Here, researchers focus on the ways in which modernity can influence microbial exposures (as one marker of biodiversity), particularly in early-life exposures. If children are indeed spending more time indoors, and less time in contact with biodiversity (compounded by the losses of surrounding biodiversity regardless of time use), there may be untold biological consequences. Throughout human Evol. ution, diverse microbial exposures have been critical to the normal training of the immune system [61]. In relation to our Evol. utionary past, rapid modernity-associated alterations to microbial exposures through antibiotic use, smaller family sizes, overuse of household “hygiene” products and dietary practices (e.g., diminished consumption of traditional fermented foods) could serve to compound the biological extinction of experience. Indeed it is now clear that the social, environmental and lifestyle dimensions of modernity are driving the propensity for systemic chronic inflammation across the life-course [62]—most linked to microbial dysbiosis—as a common denominator in the unprecedented rise in chronic non-communicable diseases globally [63,64]. 

This area of research is found under the banner of the “biodiversity hypothesis”. As stated in the World Allergy Organization position statement: “biodiversity loss leads to rEduc. ed interaction between environmental and human microbiotas. This in turn may lead to immune dysfunction and impaired tolerance mechanisms in humans” [65]. Leading experts in the field summarize it this way: “Changes in lifestyle and diet, destruction of natural environments, and urbanization threaten our natural exposure to these beneficial bacteria and thus also rEduc. e their impact on our physiology” [66]. The biodiversity hypothesis emphasizes the biological consequences of loss of experience, while the extinction of experience queries the psychological and never (or at least very rarely) the twain shall meet.

Advances in the omics technology of the exposome have already underscored that separation of the biological from the psychological is untenable [67]. With one microbial cell to match each human cell, and impressive numbers of functional microbial genes (quite capable of influencing disease risk as well as vitality [68]), humans are indeed multi-species entities [69,70]. Consider the implications of just a single non-pathogenic microbial strain influencing brain activity during social stress [71] or findings that suggest that our microbial partners could be putting their “weight” on the scale of human cognitive empathy [72] and personality [73]. Moreover, these observations open a door to narrative around very personal biological consequences of large-scale biodiversity loss [63]. 

## 7. Building Nature Relatedness

The ability to develop an emotional connection with the natural world is also, of course, dependent upon personal experience [74]. Several validated instruments capture the extent to which an individual is drawn to, has awareness of and a fascination with the natural world and its constituent parts. These scales include Nature Relatedness [75], Nature Connectedness [76] and Nature Connectivity [77]. The types of statements on these scales include items such as “My relationship with Nature is an important part of who I am” and “I enjoy digging in the Earth and getting dirt on my hands” [75,76,77]. 

**Figure 2:**
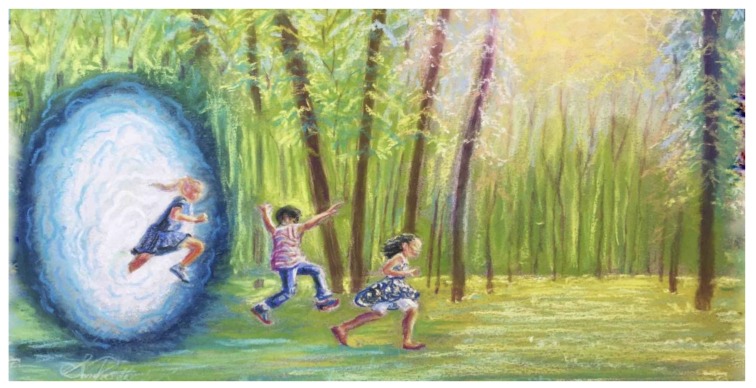
Nature connectivity in childhood improves long term physical and mental health outcomes and environmental attitudes: Regular contact with nature improves health and health behaviors, including improved physical activity, improved eating behaviors, social behaviors and lifelong nature connectedness—promoting long-term emotional well-being and pro-environmental concern as adults.

Higher scores on these scales (for convenience, collectively referred to here as nature relatedness) have been linked with health and wellbeing [78,79]. Nature relatedness is also positively associated with empathy, pro-environmental attitudes and pro-social behaviors (higher humanitarianism and lower materialism) [80,81,82,83,84], and in at least one study, nature relatedness explains a high degree of variance in children’s ecological behavior (vs. environmental knowledge alone) [85] (Figure 2). Research also shows that nature relatedness is associated with stronger connections to abstract social groups (that is, all of humanity, rather than a local social network) and distant others (such as a homeless person on the street) [86]; with this background, and separate research indicating that (at least to some degree) the health value of spending time in natural environments is predicated on baseline nature relatedness [87], it seems obvious that a greater understanding of how nature relatedness is “built” seems urgent [88,89].

However, it is our contention that the aims of planetary health require a fine-grained understanding of nature relatedness as it is intertwined with the extinction of biological experience. This necessitates a removal of the thin veneer that separates the psychological from the biological; it also requires a deeper status report on “spaceship human”. To reemphasize—we are multi-species entities with diverse biota. As each one of us moves through space and time, leaving our environmental footprints for subsequent generations, we rely upon others (including distant others, policymakers, multinational corporations and their lobbyists and marketing arms) to ensure that the spaceship human reaches its fullest potential. 

To these ends, researchers need to expand the ways in biodiversity losses are tabulated. For example, children throughout the world are increasingly dependent upon ultra-processed foods (stripped of fiber and natural phytochemicals found in whole plant foods, while inclusive of numerous emulsifiers, additives and artificial ingredients [90,91]). This is no less an extinction of experience, no less a disconnection from nature, with trans-generational biological implications due to changes in the biodiversity of the gut ecosystem [92,93,94]. While there is much to be learned, the available bench and epidemiological evidence would suggest direct lines can be drawn between extinction of biological experiences—e.g., the absence of healthy, fiber-rich foods supplanted by ultra-processed foods—and a higher risk of non-communicable diseases [62]. The extent to which the microbiota carried by (and associated with) industrialized humans has diverged in its compatibility with the human genome is an area of intense scientific scrutiny [95].

While green space and local biodiversity are associated with health and vitality [96,97,98] (and biodiversity losses/environmental degradation with ill-health [99,100,101]), these links require complex considerations. For example, closer residential proximity to green spaces and greater access to open spaces and safe, local parks, is associated with healthier dietary habits; however, this could be explained by experience through opportunity—more green spaces and parks may simply be a surrogate marker for lower density of fast-food outlets, convenience stores and an unhealthy food environment in general [102,103,104]. At this stage it is unknown if those scoring high on nature relatedness scales avoid ultra-processed foods, consume more fermented foods and/or maintain healthier dietary habits in general; however, a greater understanding of the emotional/cognitive drivers of a sustainable planetary health diet (a plant-based diet with minimal emphasis on animal products [105,106]) will require a closer look at nature relatedness.

We conclude this section with an important reminder that the bulk of the research (epidemiology, laboratory, field work and in the psychological sciences) concerning nature relatedness and the health associations linked to natural environments in general (to use the broad term) has emerged from westernized nations. Although this research has demonstrated that the value of natural environments may be particularly important for disadvantaged individuals and communities in western societies [107,108], the topic requires more research and perspectives from diverse cultures. Nature relatedness scales have been translated for use in non-western nations, and the results are consistent with findings in North America, Europe and elsewhere [109]. The available cross-cultural research demonstrates that nature relatedness is a basic psychological need [110]. 

There are many social and environmental factors that could potentially facilitate or impair the development of nature relatedness. Grotesque social inequities could easily influence time availability (e.g., demanding work hours, low wages and work conditions) and other resources that predetermine time spent connecting with the health-promoting aspects of nature or biodiversity. Moreover, it is also important to point out that “nature” is a broad term, and “exposure” to nature is often interpreted (or assumed) to be a benefit. In reality, disadvantaged individuals and communities, especially in non-Western nations, are often confronted with pathogenic microbes, lethal vectors, harsh elements and other pressures from the natural world. Despite an increasingly robust body of research on the benefits of natural environments/nature relatedness vis-a-vis health and pro-environmental behaviors, more diverse multicultural perspectives are essential for the progress of planetary health. 

## 8. Spaceships and Earthly Values

“(No one) ever seemed to acknowledge the deep irony in enlisting the help of an image that was itself the product of the very complex of economic, bureaucratic, military and technological systems they held responsible for the destruction of the environment...in missing the irony inherent in Spaceship Earth, environmentalists rendered the metaphor self-defeating” William Bryant, PhD [111].

With similar enthusiasm and hyperbole reminiscent of the 1960s, much has been written in the last several years about planned human-piloted missions to Mars—and the notion of “terraforming” the red planet into a fertile green and blue sphere [112]. Very little discussion has taken place regarding the extent to which such ideas and underwriting are a form of intellectual escapism that otherwise detract from investments in understanding the Anthropocene and its cognitive–behavioral drivers [5]. Reflecting on the origins of the term spaceship earth and the emergence of objective exposome science, we can now evaluate its utility in the contemporary lexicon.

The Spaceship Earth metaphor has not been without criticism; some have argued that the term was a product not only of the space race, but also the military industrial complex and Cold War, one which relegated nature—delicate interconnected ecosystems—to a technological artifact [113]. Anthropologist Margaret Mead was of the opinion that “the label Spaceship Earth to describe the planet is a monumental conceit” [114]. “We can think of ourselves as on something like a man-made spaceship, or we can recognize that we live within a biological system...we can treat this Planet Earth like an expendable machine, or we can recognize that our dignity is dependent upon the respect we pay to the origins of our Earthly life, and upon the responsibility we take for its preservation”, said Mead [115]. 

Dubos, in his 1969 Spaceship Earth address, leveraged the term in order to prod consideration of scientific investments and priorities:

“Modern scientists give much lip service to their social responsibilities, but in practice they behave as if they were captive of an establishment which often appears asocial...if a massive effort similar to the one represented by the National Aeronautics and Space Administration (NASA) is not soon initiated to deal with the environmental crisis, then it will be obvious that the scientific community and the governmental agencies responsible for the funding and administration of science are not as interested in human welfare as they pretend to be” [17].

Dubos was a Pulitzer-Prize-winning author; he understood the importance of metaphor in society at large, including the sciences where it can act as a catalyst in determining the types of questions scientists seek to answer; that is, questions deemed worthy of answering. Thus, as Rene Dubos said, “we must try to imagine the kinds of surroundings and ways of life we desire, lest we end up with a jumble of technologies and counter-technologies that will eventually smother body and soul” [116]; he argued that our imaginative metaphors matter. 

Today, with so many complex, interconnected problems on Earth, an emerging science seeks to understand how humans imagine a better world; that is, the science of utopian thinking. While it can be used as a pejorative (those seeking mental health reform in the mid-1800s were dismissed as “utopians”) and often confused with dystopia, utopian thinking is an imaginative process that develops ideas for change, justifies them on the basis of normative principles and places them into available scientific knowledge of the root causes of problems; the ideas can then be subjected to larger-scale critical scrutiny [117]. Utopian thinking can be an important asset to society [118].

When researchers prime individuals to engage in utopian thinking (that is, to imagine and provide a descriptive of their own utopia) it can manifest in a greater desire to take action—subjects were less satisfied with the current state of affairs and reported a rEduc. tion in system-justifying attitudes [119] (that is, the attitudes that otherwise defend, bolster and justify aspects of existing social, economic and political systems [120]). However, research suggests there may be key differences between the utopian thinking rooted in other worldly science fiction and Earthly ecology; the former, *Sci. -Fi utopia*, operates from a place of abundance and advanced technology, while the latter, *Green utopia*, is visualized from a place of balance between human development and sustainable material comfort. 

When primed with the *Green utopia*, individuals report higher levels of motivation to engage in social change activities; they also donated more to a pro-environmental charity within the research design. On the other hand, *Sci. -Fi utopia* priming did not move the needle on motivation for social change at all. The difference appears to be the experience of *participative efficacy*—stronger feelings that actions can indeed “make a difference” in achieving the aims of the visualized *Green utopia*. Researchers also found that *Green* (vs. *Sci. -Fi*) utopia was associated with positive emotions and greater interpersonal warmth and positive emotion, while still having a similar level of competence (that is, efficiency and productivity are on par with the *Sci. -Fi utopia*) [121].

Why does this matter to the current context of planetary health? Separate groups of researchers have found that individuals report higher levels of motivation to support changes to society when such changes are visualized as bringing about a society in which individuals show warmth and are collectively more benEvol. ent [122,123,124]. 

## 9. Restoration

Conservation ecology and preventive medicine share the common goals of *health* preservation and ecological balance; given the emerging links between biodiversity and human health (wellness) and vitality, as complex as they may be, the need for interdisciplinary approaches to preserving health at all scales (species, habitats and ecosystems) is now apparent. In addition to conservation/prevention, the sheer scale of environmental destruction and biodiversity losses—and the pace at which they are occurring—requires proactive steps to remediate/restore degraded ecosystems (in much the same way that clinical interventions attempt to address NCDs). To this end, concepts of de-extinction and rewilding have entered mainstream academic discourse. 

Briefly, de-extinction utilizes advances in synthetic biology, genetic engineering and reproduction technologies to revive currently extinct species (or hybrids inclusive of traits of extinct species—e.g., woolly mammoth genes incorporated into Asian elephant embryos) and reintroduce them to areas that might resemble their former habitats [125]. Rewilding was originally defined as restoring “Big Wilderness” by focusing on a top-down approach whereby keystone species and large animals are provided ample space and corridors for sustainable movements [126]; today, rewilding is defined more broadly as the “restoration of wildness” [127] wherein wildness refers to the autonomy of natural process that are characteristic of scales ranging from vast wilderness areas to vacant lots in an urban environment. Restoration is likely a more suitable term, especially since the endgame of “rewilding” and “de-extinction”, insofar as planetary health is concerned, is to return the Earth’s natural systems to a healthier condition. 

De-extinction has been a contentious topic, particularly in the context of bioethics; discourse concerning the ethical implications of restoring species (most notably the wooly mammoth and passenger pigeon) can be found elsewhere [128,129]. The extent to which de-extinction of select species can resurrect the ecology of the extinct species (that is, a beneficial influence on broad ecological dynamics) is in the domain of functional ecology [130]; de-extinction forces many questions for the social sciences, including “what kind of nature does de-extinction seek to make?”, “what are the political economies of de-extinction?” and “who (humans and other species) accumulates gains/losses with this kind of nature?” [131].

Whether or not a new hybrid of the woolly mammoth roams in Pleistocene Park (as planned within the next decade), the technological tools of de-extinction—genomics in particular [132]—will almost certainly apply to smaller scale rewilding. Although the “ideal” human gut microbiome is elusive, and dysbiosis is a relative term [133], microbes that appear to be functionally extinct in Westernized populations (yet found in the few remaining hunter–gatherer and other traditional-living groups), as well as soil-based organisms, are candidates for rewilding of the gut/skin microbiota, certainly with an eye toward health preservation [93,134,135]. In addition, microbiota-based interventions within homes, workplaces and urban green spaces—micro-rewilding—offer potential solutions to combat the prevailing rEduc. tion in contact with biodiversity [136,137,138]; already, sophisticated DNA-based research is providing important data concerning the ways in which woodland ecosystem restorations projects might enhance human exposure to immune-modulating microbial diversity [139].

## 10. De-Extinction of Experience, Psychobiological Restoration: Made Personal

While science and technology march forward with innovate fixes to remediate and restore ecosystems to health at scales of person and place, it must be acknowledged that human cognition and behavior has directed us to the Anthropocene; unless we approach solutions through the lens of the psyche (individual and collective), the fulfillment of rewilding potential will likely be diminished. Put another way, the potential for success with rewilding efforts will be enhanced by a greater understanding of nature relatedness and the psychobiological underpinnings of human connections to the natural world (and how/why we seem so willing to allow for its degradation). De-extinction of experience with the natural world, even with its diminished levels of biodiversity, should be prioritized. 

Insofar as we are unwilling to consider that rewilding of our value systems requires at once deeper connections with the natural world, and relinquishing the extreme production/consumption characteristic of the Anthropocene, the re-introduction of the wooly mammoth and washing with soil-based soaps is unlikely to save us from ourselves. Rewilding, in our opinion, requires a change in the way we view systems (e.g., food, Educ. ation and political) that either compromise health at the scales of the person, place and planet, or ignore the development of an eco-health psyche. 

Rewilding planetary health values requires understanding the ways in which neoliberal ideologies drive global materialism, environmental destruction and grotesque consumption, placing the sole responsibility for health and healthy behaviors on the individual while undermining their capacity to do this [140,141]. These ongoing competing interests are unsustainable. Efforts to achieve “personalized medicine” will ultimately fail if they are counter to the economic forces driving inequity, social injustice and proliferation of grey space. Individual interests and responsibilities must be facilitated by the interests of society at large for personalized medicine (wellness) to be truly meaningful. This calls for reinvigorating concepts of “high-level wellness”, in which not only individuals reach their potential, but the interests of individuals, societies and the environment are aligned, with the recognized co-benefits of mutualism [142,143]. 

Fundamentally, these perspectives also require a more “whole person” functional approach to promoting health through nutrition, lifestyle and environment as a greater priority—with increasing evidence that this is important and effective in improving health-related quality-of-life [144].

This must be in tandem with a “long view” that considers not only a life course approach, but also the transgenerational implications. Volumes of developmental origins of health and disease (DOHaD) research emphasizes that health through young adulthood, middle age and through the aging process is set on a trajectory very early in life. Moreover, it is well known that environmental exposures (including pollutants, adverse nutrition and a variety of stressors) in one generation mediate “heritable” epigenetic effects on DNA that can influence the phenotype and disease predisposition of the next generation(s) [145]. The same research has revealed that, in addition to genetics, early environmental conditions contribute to “thrifty phenotypes” that store calories more than those with calorie burning “spendthrift phenotypes”—providing yet another dimension to personalized medicine [146,147]. It also reinforces that there are multifaceted reasons for individual differences in response to lifestyle interventions and why future preventive approaches are likely to be more effective if they are individualized according to phenotype (patterns of gene expression).

Of specific relevance to ecological discussions, included in DOHaD findings, is intriguing research that suggests that non-pathogenic microbes (and other aspects of biodiversity) in early life have multisystem consequences through effects on immune development [148]. One of the more fascinating dimensions of this has been revelations that this might even influence subsequent brain development and mental health [149,150,151,152]. When paired with separate research on access/residential proximity to natural environments and healthy birth outcomes (again, setting life-course health) [153,154], the psychobiological implications of rewilding loom large. While there is much to learn, it seems obvious that rewilding can be viewed as part of a larger effort to start undoing the extinction of experience, re-imagining an early-life Educ. ation that fosters empathy for each other, and a planet in crisis. Therefore, narratives around individual efforts towards improving personal ecology (through nutrition, lifestyle behaviors and interventions such as probiotics when indicated) must ultimately occur within a societal narrative with the same goals across all sectors—ideally through a shared overarching perspective that assists in shifting normative value systems.

## 11. Conclusions

In an age of destruction and polarization on a never-before-seen scale, it is, more than ever, important to be reminded of the calls for civility and good citizenship on “Spaceship Earth” [17]—at the time of the first moon landing in 1969 as we saw our exquisitely beautiful, fragile planet from across the void of space. It was a moment to be reminded of calls for “civilization” during the Enlightenment of the eighteenth century—the quest for a universal humanity that valued moderation, self-regulation, humane laws, a high level of purpose, decency in conduct and limitations on war. Even then, it was clear that the “health of human civilization” necessitated a frameshift in attitudes, intentions, emotions, ideals, values and, especially, behaviors [155]. While *all* of these elements do sit in the domain of individuals, this should be harnessed to add weight to the call for greater responsibility by global leaders, dominant groups and influential organizations to apply both science and humanity to promote planetary health—and call out efforts to subvert this [156,157,158,159,160,161,162,163] to restore hope and public trust [164].

Linguistics research leaves little doubt that human beings interpret the world around us through metaphors [165,166]; indeed, even common metaphors have been found to be useful as a means to enhance the pursuit of goals [167]. Hence, discussions of metaphor in the context of planetary health are salient. Spaceship Earth has been used as a metaphor for over a century, gaining popularity for its utility in promoting environmental awareness during the technologically driven space race of the 1960s.

Today, as tremendous resources are directed at planning for crewed flights and colonization on Mars (and “terraforming” the red planet with life) [112,168,169], and as social scientists remind enthusiasts of the more pressing priorities here on Earth, the Spaceship Earth metaphor is reentering the discourse [170]. This represents a new opportunity to rewild an old, but salient, metaphor. The keyword in the metaphor is *Earth*. While technology and scientific exploration are vital to solving the interconnected grand challenges of our time, the fullest potential of our investments in such endeavors will require an understanding of the developmental origins of an individual and collective “Earth-oriented, eco-health psyche”, one which comprehends why/how de-extinction and rewilding have entered the scientific lexicon.
